# Antiprogramed cell death‐1 therapy with microspheres for metastatic liver tumors

**DOI:** 10.1002/jgh3.12213

**Published:** 2019-06-24

**Authors:** Hiroteru Kamimura, Nobutaka Takeda, Takashi Owaki, Takeshi Mizusawa, Takahiro Iwasawa, Satoshi Ikarashi, Satoru Hashimoto, Masaaki Takamura, Shuji Terai

**Affiliations:** ^1^ Division of Gastroenterology and Hepatology Niigata University Graduate School of Medical and Dental Sciences Niigata Japan

**Keywords:** anti‐programmed cell death‐1 therapy, melanoma, metastatic liver tumor

## Abstract

Anti‐programmed cell death‐1 therapy with microspheres is an effective treatment for metastatic liver tumors.

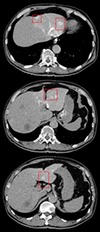

Immunotherapy is an effective treatment for many cancers that require surgery, radiotherapy, and chemotherapy.^1^ The use of antibodies that specifically block programed cell death‐1 (PD‐1) was approved for the treatment of melanoma in 2014.^2^ Combining immunotherapy with other therapies, including locoregional therapy (e.g. transarterial embolization), is an innovative field of clinical investigation for cancer treatment. With the advent of immunotherapy for hepatocellular carcinoma, interest in understanding how immunotherapy may be best combined with locoregional therapies is increasing.^3^ Advanced melanoma presents a significant therapeutic challenge to clinicians. Many therapies for metastatic melanoma are limited by low response rates, severe toxicities, and/or relatively short response durations.^4^ Here, we report a case of partial remission and tumor regression after antiprogramed cell death‐1 (anti‐PD‐1) therapy with microspheres.

A 67‐year‐old man presented to our division for additional treatment of multiple liver metastases. Six years ago, he was diagnosed with left uveal melanoma, and his eyeball was enucleated. Four years ago, liver metastasis was detected in S6, and segmental resection was performed. Pathological examination confirmed the diagnosis of delayed metastasis from the uveal melanoma. Two years ago, the liver tumor had grown multifocally (Fig. 1a); hence, anti‐PD‐1 therapy was initiated. However, the metastatic liver tumor was progressive; thus, we performed additional therapy with transarterial embolization using microspheres, sized 100–300 μm, in the left lobe, selectively. After a 3‐month follow‐up, computed tomography demonstrated a necrotic cyst and partial remission of the tumor in the left lobe (Fig. 1b). The treatment effects on the tumors persisted even after 1 year. Anti‐PD‐1 therapy was continued basically but was stopped for 2 weeks during the embolization therapy. No major complications occurred. Selective hepatic artery embolization with microspheres is a safe treatment for patients with metastatic melanoma.

**Figure 1:**
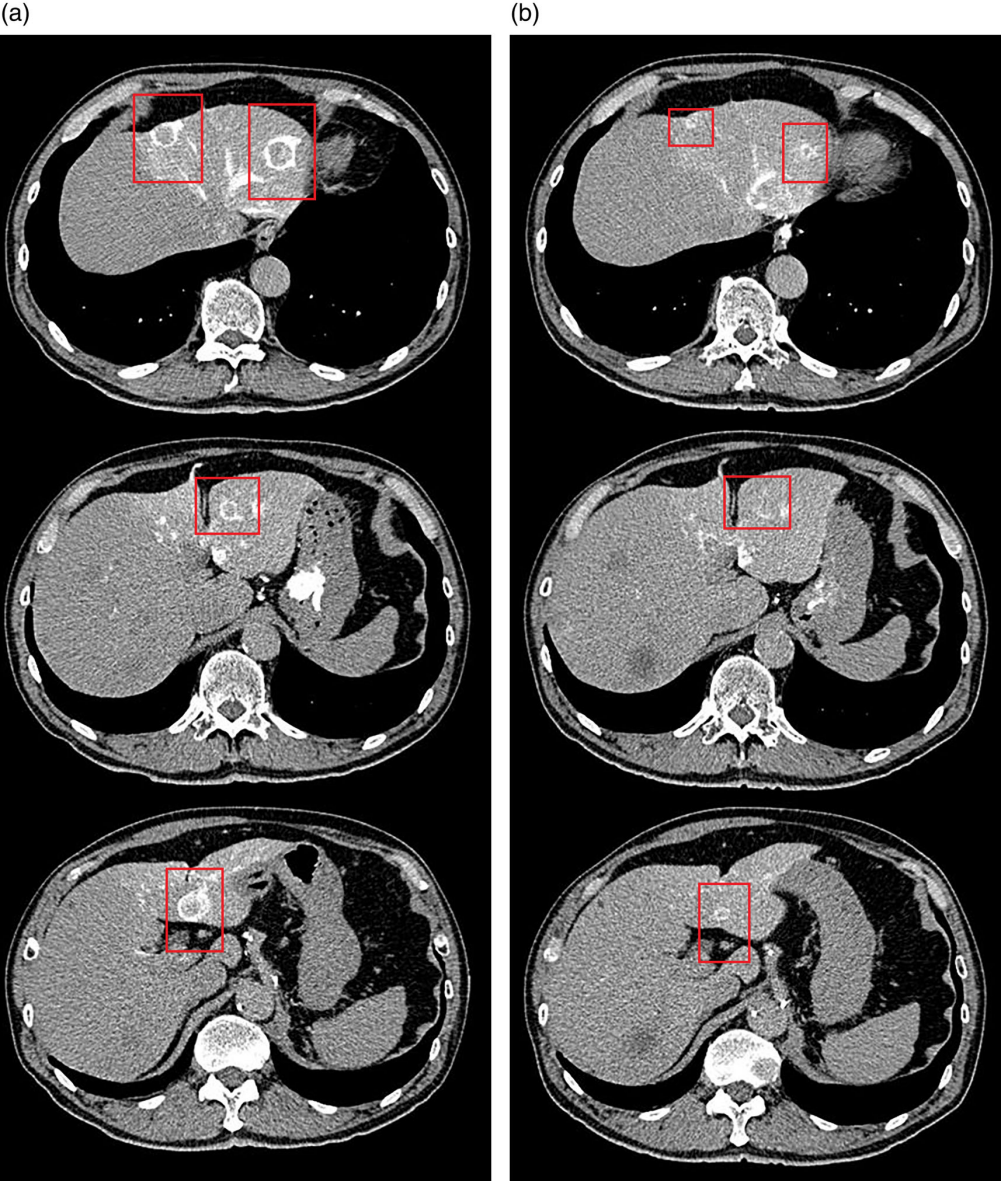
(a) Computed tomographic angiogram showing a high‐density area in the left lobe in computed tomography during left hepatic arteriography (CTLHA) in the second phase. (b) The second‐phase CTLHA demonstrated a necrotic cyst and tumor regression of the tumor in the left lobe after a 3‐month follow‐up treatment.
